# Retraction: Violation of the ultrastructural size principle in the dorsolateral prefrontal cortex underlies working memory impairment in the aged common marmoset (Callithrix jacchus)

**DOI:** 10.3389/fnagi.2026.1980330

**Published:** 2026-09-03

**Authors:** Frontiers Editorial Office

The authors retract the article cited above.

Following publication, the authors contacted the Editorial Office to request the retraction of the cited article, stating that an indexing error affected the dataset for the aged impaired marmoset, causing presynaptic boutons to be misaligned with their corresponding presynaptic mitochondria in approximately half of the records for that animal. Reanalysis of the corrected data showed that the reported correlation between mitochondrial size and synapse size—described in the original article as abolished in the aged impaired animal—was not supported, materially altering the article's central findings and conclusions. The findings and conclusions reported in the article are no longer supported by the corrected data. An investigation was conducted in accordance with Frontiers' policies that confirmed this; therefore, the article has been retracted.

This retraction was approved by the Chief Editors of Frontiers in Aging Neuroscience and the Chief Executive Editor of Frontiers. The authors agree to this retraction.

